# Optimizing Choice of Skin Surrogates for Surface-Guided Stereotactic Body Radiotherapy of Lung Lesions Using Four-Dimensional Computed Tomography

**DOI:** 10.3390/cancers16132358

**Published:** 2024-06-27

**Authors:** Vanda Leipold, Ivana Alerić, Mihaela Mlinarić, Domagoj Kosmina, Fran Stanić, Mladen Kasabašić, Damir Štimac, Hrvoje Kaučić, Giovanni Ursi, Karla Schwarz, Igor Nikolić, Denis Klapan, Dragan Schwarz

**Affiliations:** 1 Faculty of Medicine, Josip Juraj Strossmayer University of Osijek, 31000 Osijek, Croatia; 2 Specialty Hospital Radiochirurgia Zagreb, 10431 Sveta Nedelja, Croatiadomagoj.kosmina@radiochirurgia.hr (D.K.); hrvoje.kaucic@radiochirurgia.hr (H.K.);; 3 Bitwise Solutions d.o.o., 10000 Zagreb, Croatia; 4 School of Medicine, University of Zagreb, 10000 Zagreb, Croatia; 5 School of Medicine, University of Mostar, 88000 Mostar, Bosnia and Herzegovina; 6 Faculty of Dental Medicine and Health Osijek, 31000 Osijek, Croatia; 7 Faculty of Medicine, Juraj Dobrila University of Pula, 52100 Pula, Croatia

**Keywords:** four-dimensional computed tomography (4DCT), stereotactic body radiotherapy (SBRT), image-guided radiotherapy (IGRT), surface-guided radiotherapy (SGRT), region of interest (ROI)

## Abstract

**Simple Summary:**

Surface tracking based on optical cameras is often used to reduce the imaging dose while maintaining precision during stereotactic ablative body radiotherapy. A region of interest (ROI) on a patient’s skin is selected and monitored using harmless visible light in real time. Relying on the correlation between the skin and lesion respiratory motion, this region of interest is used as a surrogate for the tumor respiratory motion. In this study, the thoracic and abdominal skin region is segmented into nine smaller regions. For each of them, the respiratory motion magnitude and its correlation with a small structure in the lower lung (representing a lung lesion) are measured. Using the data provided by this study, a clinician can make an informed decision about which ROI to track when treating lower lung lobe lesions.

**Abstract:**

Image-guided radiotherapy supported by surface guidance can help to track lower lung lesions’ respiratory motion while reducing a patient’s exposure to ionizing radiation. However, it is not always clear how the skin’s respiratory motion magnitude and its correlation with the lung lesion’s respiratory motion vary between different skin regions of interest (ROI). Four-dimensional computed tomography (4DCT) images provide information on both the skin and lung respiratory motion and are routinely acquired for the purpose of treatment planning in our institution. An analysis of 4DCT images for 57 patients treated in our institution has been conducted to provide information on the respiratory motion magnitudes of nine skin ROIs of the torso, a tracking structure (TS) representing a lower lung lobe lesion, as well as the respiratory motion correlations between the nine ROIs and the TS. The effects of gender and the adipose tissue volume and distribution on these correlations and magnitudes have been analyzed. Significant differences between the ROIs in both the respiratory motion magnitudes and their correlations with the TS have been detected. An overall negative correlation between the ROI respiratory magnitudes and the adipose tissue has been detected for ROIs with rib cage support. A weak to moderate negative correlation between the adipose tissue volume and ROI-to-TS respiratory correlations has been detected for upper thorax ROIs. The respiratory magnitudes in regions without rib support tend to be larger for men than for women, but no differences in the ROI-to-TS correlation between sexes have been detected. The described findings should be considered when choosing skin surrogates for lower lung lesion motion management.

## 1. Introduction

The treatment of primary and metastatic lung tumors requires a multidisciplinary, individualized approach [1,2]. One of the techniques that allows for a high level of individualization is surface-guided radiotherapy (SGRT). There has been a surge in the clinical application of optical surface guidance in radiotherapy over the past decade [3,4,5,6,7]. Surface guidance (SG) is used to reduce the imaging dose while maintaining precision in tumor targeting during stereotactic body radiation therapy (SBRT). A set of optical cameras detecting harmless, non-ionizing light is used to track a region of skin in a three-dimensional space and in real time. Since there is a correlation between the skin and lesion motion, it is possible to infer the position of the lesion based on the position of the monitored skin region. In this way, real-time information on the tumor location is acquired without additional X-ray imaging. SG is used for patient setup and incidental motion detection and has been shown to provide the same or better results when used for patient positioning and motion tracking as compared to traditional methods (lasers and markers or tattoos) [8,9,10,11,12,13]. 

Surface guidance is also used for the gating of tumors subject to respiratory-induced motion—for instance, using breath hold (BH) or phase gating techniques. A therapeutic beam is delivered while the patient’s skin is within a certain tolerance window, since it can be verified that while the skin is within this window, the lesion remains within the planned volume. Optical surface guidance is not only efficient for the gated treatment of targets near the skin surface—for example, the breast [14,15,16]—but also for the gated treatment of intrathoracic and abdominal targets, such as lung or liver lesions [17,18,19,20,21,22,23].

It has been demonstrated, however, that not all skin segments are equally suitable surrogates for lesion motion management. 

Bry et al. noted that different shapes and sizes of ROIs selected for the surface-guided treatment of patients immobilized using open-face masks can affect the incidence of false positional corrections reported by surface guidance systems [24].

Sauer et al. have reported that different placements and sizes of ROIs used during breast radiotherapy in the breath hold (BH) technique can significantly affect the success of motion management [25]. 

Zeng et al. analyzed patients treated for left-sided breast cancer using surface guidance and the breath inspiration technique. They found that different ROIs should be used when treating patients with different breathing behaviors (thoracic vs. abdominal breathing) [26]. 

Laaksomaa et al. compared three different ROIs used for patient setup during the surface-guided radiotherapy of the breast and found a small difference between them [27]. 

Cui et al. [28] and Chen et al. [29] have both developed ROI selection algorithms for surface-guided breast radiotherapy.

Yulin Song et al. analyzed several skin markers placed in areas of the abdomen, used as surrogates for thoracic lesions treated in the breath hold (BH) technique, and found that the markers inferior to the rib cage had a better correlation with the diaphragm movement compared to those placed in the area of the rib cage [30]. 

Aside from ROI selection, Jiateng Wang et al. noted that the correlation between the tumor and skin motion can vary greatly between tumors and patients, implying that, when planning lung and liver SBRT motion management, individual patient and tumor characteristics should always be considered [31]. 

The existing studies on the selection of optimal skin surface surrogates for lung lesion tracking are primarily focused on selected small regions of the thorax or the abdomen and the marker-to-lesion, rather than ROI-to-lesion, respiratory motion correlation. Furthermore, while focusing on the respiratory motion correlation, the skin respiratory motion magnitudes are often neglected. Since optical surface monitoring systems with beam-hold allow beam-on only while the skin segment is within a tolerance window, the relative magnitude of respiratory motion within this window should also be taken into consideration. 

Heinzerling et al. found that surface guidance may be a less reliable patient setup tool when used for patients with increased volumes of adipose tissue [32]. It remains unclear how the adipose tissue distribution affects the respiratory ROI magnitudes and ROI-to-lesion correlations. 

In this study, four-dimensional computed tomography (4DCT) images of patients acquired for the purposes of treatment planning were used to generate correlation maps between the patient’s skin and a tracking structure (TS) in the lower right lung lobe. Respiratory magnitude maps of the whole thorax and abdomen were also generated and used to determine the areas of skin best correlated to the lower lung lobe structure motions. Different ROIs were compared by correlation and amplitude to determine those best suited to be used as surrogates for the lower lung lobe structures. Significant differences between the ROIs were detected, in terms of both the respiratory magnitudes and the ROI-to-TS respiratory correlations.

Possible connections between the respiratory ROI magnitudes and ROI-to-TS correlations, on one hand, and the patient’s gender, height, body mass index (BMI) and subcutaneous and visceral adipose tissue volumes, on the other hand, were analyzed.

## 2. Materials and Methods

The 4DCT images of 57 patients (31 female and 26 male), with an average age of 68 years (range 43–91) were used in this study. The images were acquired at our institution for the planning of SABR treatments for oligometastatic lesions arising from colorectal (37), pancreatic (11), kidney (5) and genitourinary carcinoma (4).

The 4DCT images with artifacts in the area of the lower lung, as well as those with a skin structure that did not encompass the patient’s skin from the upper half of the sternum to below the umbilicus (below L4), were not included in this study. Patients whose breathing was affected by paresis of the diaphragm were not included in this study.

**Figure 1:**
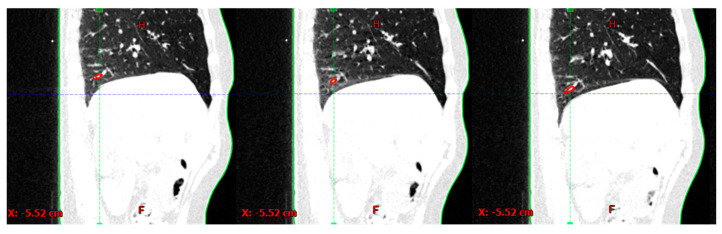
An example of a tracking structure (TS) (red) and skin (green) in three out of ten 4DCT phases.

Using the Eclipse treatment planning system (TPS) (Varian Medical Systems, Palo Alto, CA, USA), a segment of a blood vessel in the lower right lung lobe, i.e., the target structure (TS), and the patient’s skin (skin structure) was generated on the first of the 10 4DCT phases and propagated to the other phases; see Figure 1.

**Figure 2:**
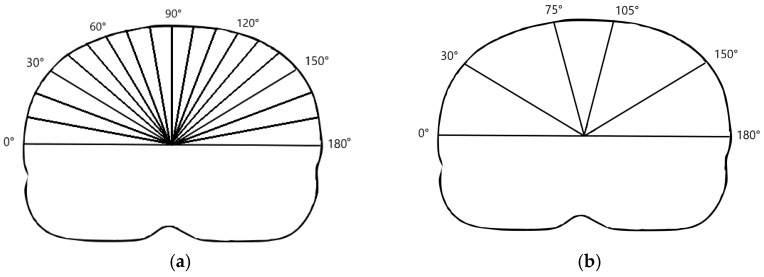
(**a**) Radial beams originating from the craniocaudal axis. Only beams at intervals of 10° are shown. (**b**) Right, central and left skin regions corresponding to beam angles in the ranges of 30°–75°, 75°–105° and 105°–150°, respectively.

Using an in-house-developed computer program, a centroid for the skin contour was determined at each slice. The least-squares method was applied to the skin centroids in order to generate a central craniocaudal axis. For each of the CT slices (1 mm thickness), a set of radial beams at 1° intervals from 0° to 180° (total of 180 beams), connecting the axis to the skin structure, was generated; see Figure 2a. The beam length variations between respiratory phases were used as a measure of the skin respiratory motion. The TS centroid coordinate variations in the craniocaudal direction between respiratory phases were used as a measure of TS motion. Only the craniocaudal direction was considered since it is the predominant motion direction for lower lung lobe structures. 

The skin was divided into the left, right and central regions based on the angle of the radial beams, corresponding roughly to the regions to the left, between and right of the parasternal lines, respectively, as shown in Figure 2b.

**Figure 3:**
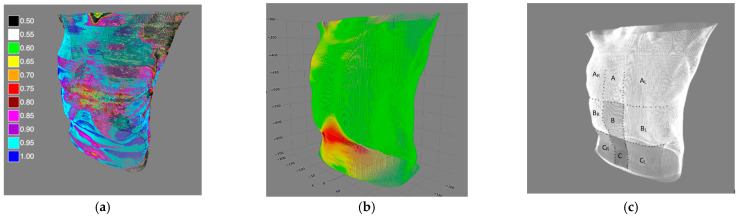
(**a**) An example of a skin-to-TS correlation map. Pearson’s R values are color-coded from the lowest values (R < 0.5) in black to the highest values (R > 0.95) in deep blue. (**b**) An example of an intensity map. Skin areas with the largest excursions are marked in red, and areas with the lowest excursions are marked in green. (**c**) Regions of interest are divided horizontally by parasternal lines and vertically by the xipho-sternal line and subcostal plane into regions A, A_R_, A_L_, regions B, B_R_, B_L_, and regions C, C_R_, C_L_.

The Pearson correlation between the beam lengths and the TS’s craniocaudal coordinates in 10 4DCT phases was computed for each of the beams, generating a skin correlation map (Figure 3a). The beam length variation magnitudes (i.e., maximum skin excursions) were used to generate a respiratory magnitude map (Figure 3b).

The average respiratory magnitudes and the correlations with the TS respiratory motion were computed for nine regions, as shown in Figure 3c. The results were compared between regions using the Friedman test and Dunn–Bonferroni post hoc test. 

For a single CT slice, abdominal adipose tissue estimation was performed at the level of L4 [33,34]. The subcutaneous adipose tissue (SAT), visceral adipose tissue (VAT) and total adipose tissue (TAT) surfaces were measured, and their relative values to the total CT slice surface were computed. To evaluate the connection between the adipose tissue, the BMI, the patient’s height and the ROI respiratory magnitudes and their correlations with the TS, Spearman’s Rho was used.

Differences in the ROI respiratory magnitudes and ROI-to-TS correlations between men and women were tested using the Mann–Whitney U-test.

## 3. Results

Respiratory motion skin magnitude maps and skin-to-TS-Pearson’s-R correlation maps were generated. The median magnitude and correlations were computed for nine skin ROIs. The magnitude of respiratory motion was measured for all target structures. 

### 3.1. Magnitude and Correlation Maps

**Figure 4:**
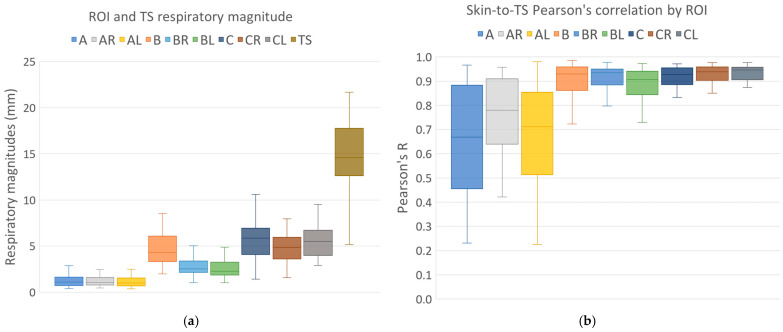
(**a**) Respiratory magnitudes of different regions of interest (ROIs) and TS; (**b**) ROI-to-TS respiratory correlations. The box plot shows the medians, the lower and upper quartiles and the lower (and upper) one-and-a-half interquartile ranges.

The respiratory magnitudes and ROI-to-TS correlations for different regions are shown in Figure 4a and Figure 4b, respectively. The differences in the respiratory magnitudes assessed using Friedman’s nonparametric test for repeated measurements were found to be significant (Chi^2^(8) = 372.2; *p* < 0.001). The post hoc Dunn–Bonferroni test results for the differences in the respiratory magnitudes are presented in Table 1. 

The differences in the ROI-to-TS correlations between different ROIs were also tested using Friedman’s nonparametric test for repeated measurements and were found to be significant (Chi^2^(8) = 221.7; *p* < 0.001). The post hoc Dunn–Bonferroni test results for the differences in the ROI-to-TS correlations are shown in Table 1.

### 3.2. Magnitude and Region-of-Interest-to-Tracking Structure (ROI-to-TS) Correlations Related to Patient’s Sex

The respiratory magnitudes and ROI-to-TS correlations are presented in Table 2. No difference in the ROI-to-TS correlation between the ROIs of patients of different sexes has been detected.

The small effect size in the differences between the respiratory magnitudes of ROIs A, A_R_ and A_L_ for men and women, with the magnitudes tending to be larger for women, was not statistically significant.

A medium effect size in the respiratory magnitude differences between men and women, with the magnitudes tending to be larger for men, was detected in regions B (U = 253; z = −2.4; *p* = 0.02; effect size *r* = 0.32), B_R_ (U = 266.5; z = −2.19; *p* = 0.03; *r* = 0.29), C (U = 255, z = −2.4; *p* = 0.02; effect size *r* = 0.31), C_R_ (U = 258, *p* = 0.02, effect size *r* = 0.31) and C_L_ (U = 175, z = −3.65; *p* < 0.001; effect size *r* = 0.48).

The difference in the tracking structure respiratory magnitudes between men and women was tested using a *t*-test for independent samples, but no significant difference was found (t(55) = −1.12; *p* = 0.27; 95% confidence interval [−3.52, 1.00]; Cohen’s d = 0.30).

### 3.3. ROI Magnitudes Related to Patient’s Adipose Tissue, BMI and Height

**Figure 5:**
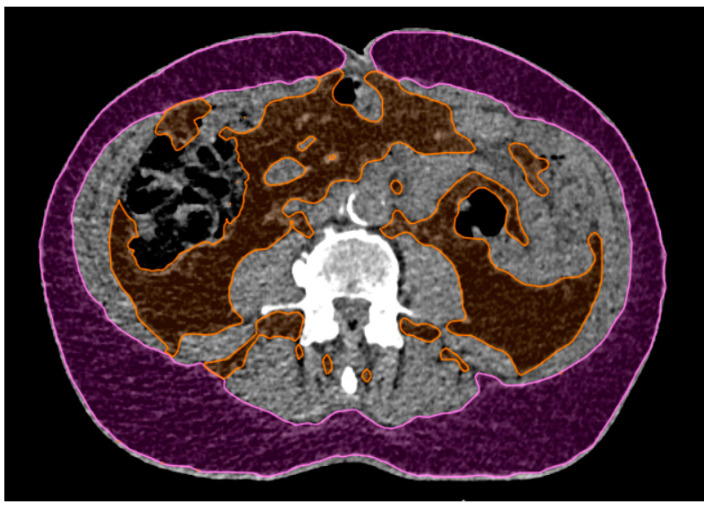
An example of subcutaneous (magenta) and visceral (orange) adipose tissue delineated on a single CT slice at the level of L4. Areas of the delineated surfaces relative to the total slice surface area were used to compute the subcutaneous, visceral and total adipose tissue percentages (SAT%, VAT% and TAT%, respectively).

The adipose tissue was measured on a single CT slice at the level of L4. The tissue in the range of −20 to −150 Hounsfield units was delineated and the total adipose tissue (TAT) was separated into visceral (VAT) and subcutaneous (SAT) adipose tissue, as shown in Figure 5. The total slice surface area was also measured. The percentage of total (TAT%), visceral (VAT%) and subcutaneous (SAT%) adipose tissue was determined. For the body mass index, the patient data collected at the time of 4DCT acquisition were used.

An overall negative correlation between the adipose tissue percentage and ROI respiratory magnitudes has been detected, as seen in Table 3. 

Assessed over the total patient sample, a weak to moderate negative correlation between the VAT% and ROI respiratory magnitudes was observed in regions A (Rho = −0.28; *p* = 0.04), A_R_ (Rho = −0.41; *p* = 0.002) and A_L_ (Rho = −0.41; *p* = 0.002).

A moderate negative correlation between the TAT% and ROI respiratory magnitudes was observed in regions A_R_ (Rho = −0.39; *p* = 0.003) and A_L_ (Rho = −0.43; *p* = 0.001) and regions B (Rho = −0.46; *p* < 0.001), B_R_ (Rho = −0.33; *p* = 0.01) and B_L_ (Rho = −0.30; *p* = 0.03). 

A moderate negative correlation between the BMI and ROI respiratory magnitudes was observed in regions A (Rho = −0.31; *p* = 0.02), A_R_ (Rho = −0.35; *p* = 0.008) and A_L_ (Rho = −0.42; *p* = 0.001); regions B (Rho = −0.42; *p* = 0.001), B_R_ (Rho = −0.28; *p* = 0.03) and B_L_ (Rho = −0.28; *p* = 0.03); and region C _L_ (Rho = −0.34; *p* = 0.01).

A weak and not statistically significant connection between the patient height and ROI respiratory magnitudes was detected using this sample size. 

### 3.4. ROI-to-TS Correlation Related to Patients’ Adipose Tissue, BMI and Height

For the adipose tissue and ROI-to-TS correlation, the connection was not an overall negative one, as seen in Table 3. For ROIs in the upper part of the thorax, a negative correlation between the adipose tissue and ROI-to-TS correlation was detected. The effect was low to moderate in strength when the total population was considered, but it varied between ROIs and between men and women. 

When men and women were assessed cumulatively, a moderate negative correlation between the ROI-to-TS respiratory correlation and VAT% was observed for regions A (Rho = −0.38; *p* = 0.004), A_R_ (Rho = −0.34; *p* = 0.01), A_L_ (Rho = −0.34; *p* = 0.01), B_R_ (Rho = −0.33; *p* = 0.01) and B_L_ (Rho = −0.30; *p* = 0.02). 

A moderate negative correlation between the ROI-to-TS respiratory correlation and TAT% was observed for regions A (Rho = −0.35; *p* = 0.007), A_R_ (Rho = −0.39; *p* = 0.003) and A_L_ (Rho = −0.43; *p* = 0.001).

A moderate negative correlation between the BMI and ROI-to-TS respiratory correlation in regions A (Rho = −0.33; *p* = 0.01), A_R_ (Rho = −0.31; *p* = 0.02) and A_L_ (Rho = −0.33; *p* = 0.01) was observed.

A low, positive and not statistically significant connection between the patient height and ROI-to-TS respiratory correlations was detected using this sample size. 

## 4. Discussion

This study found that all of the nine thoracic and abdominal ROIs showed a strong correlation with the craniocaudal respiratory motion of the anatomical landmark inside the lower lung lobe, although the correlation was poorer for the upper thoracic ROIs. These findings are in accordance with previously conducted studies that reported a strong correlation between a single skin region or marker (located near the xiphoid process) and anatomical landmarks/lesions located in the thorax and/or abdomen [20,31,35,36]. These findings are also in accordance with the study of Yulin et al. [30], who examined the respiratory correlations between several abdominal skin regions and the liver dome and reported a stronger correlation in areas without rib support.

However, along with the skin-to-lesion correlations, the skin respiratory magnitudes also need be considered when setting up skin surface tolerances for gated treatment [3]. This study found that the respiratory motion magnitudes of both the tracking structure and skin varied greatly between patients and ROIs, further reinforcing the potential importance of regions B, C, C_R_ and C_L_ for surface tracking in gated free-breathing patients, as well as the importance of an individualized approach when treating these patients.

Although the sample size was limited, a moderate negative correlation between the patient body mass index, visceral adipose tissue and ROI-to-TS correlations was detected, especially for the ROIs in the upper thoracic region and those with rib support. This is in accordance with Heinzerling et al.’s [32] report that the initial setup accuracy and intrafraction patient position monitoring based on surface guidance deteriorate as the BMI of the patient increases. In addition, a moderate negative connection between the BMI, visceral adipose tissue volume and ROI respiratory magnitudes was detected in the upper thoracic regions.

The effects of the patient height on the ROI respiratory magnitudes were positive, but the effect size was small and not statistically significant with the sample size used. 

In accordance with Wang et al. [31], no differences between men and women in the ROI-to-TS correlation maps were detected. The ROI respiratory magnitudes, however, were overall higher for men than for women, especially in the lower abdominal regions.

### Study Limitations

There are several limitations to this study. As with Song et al. [30], all of our findings were based solely on 4DCT images. They did not encompass possible changes in breathing amplitudes and patterns during treatment, which a study conducted on retrospective treatment session data could include. On-couch monitoring and verification during an SGRT session, as conducted by Kiser et al. [22], could further add to the value of this study. Phantom-based validation, similar to that of Paolani et al. [20], used to verify their model, could be used to evaluate the connection between the correlations, respiratory magnitudes and dosimetric quality of gated plans.

In addition to this, effects of different sizes were measured in this study. The sample size of 57 patients was large enough to achieve an appropriate significance level for the differences in the respiratory magnitudes and ROI-to-TS correlations between different ROIs. However, a larger sample size could provide stronger evidence, especially regarding the effects of the adipose tissue volumes, patient height and body mass index on the ROI behavior. The findings of this study also suggest possible differences in the adipose tissue on correlation and magnitude maps between men and women, but, due to the aforementioned size effect and sample size, this requires further investigation. 

Finally, this study tracked a structure in the lower lung lobe. Its findings may not apply to lesions located elsewhere—for example, apical or central lesions.

## 5. Conclusions

Since all forms of surface tracking use a threshold within which the skin surrogate needs to remain for the beam to be applied, the breathing magnitude, along with the respiratory motion correlation between the skin region and lesion, should be key when choosing the skin surrogate. If the skin ROI is strongly correlated with the target structure, but its respiratory motion magnitude is comparable to or smaller than the gating window, it may not be a good choice for gated SBRT. The results of this study imply that regions A, A_R_ and A_L_ should only ever be tracked to detect incidental motion, as regions B_R_, B_L_ and especially B, C, C_R_ and C_L_ are more suitable skin surrogates for the gated treatment of lower lung lobe lesions, both in terms of the respiratory magnitudes and the ROI-to-TS correlations. This could be especially pronounced for patients with a high body mass index and large volumes of visceral adipose tissue.

Due to the large variations observed between the patients, the choice of skin surrogate, and especially the appropriate tolerances for a gated treatment, should be assessed individually. 

It should also be noted that these findings relate to the free-breathing technique only—correlation maps and magnitude maps for patients treated using the breath hold technique may behave differently and require further investigation.

## Figures and Tables

**Table 1:** Dunn–Bonferroni post hoc test for differences in respiratory magnitude.

ROI	ROIs with Different Respiratory Magnitudes	Adjusted *p*	ROIs with Different Skin-to-TS Correlations	Adjusted *p*
A	B, B_R_, B_L_, C, C_R_, C_L_	<0.001	B, B_R_, B_L_, C, C_R_, C_L_	<0.001
A_R_	B, B_R_, B_L_, C, C_R_, C_L_	<0.001	B, B_R_, B_L_, C, C_R_, C_L_	<0.001
A_L_	B, B_R_, B_L_, C, C_R_, C_L_	<0.001	B, B_R_, B_L_, C, C_R_, C_L_	<0.001
B	A, A_R_, A_L_, B_R_, B_L_	<0.001; <0.002 for B_R_	A, A_R_, A_L_	<0.001
B_R_	A, A_R_, A_L_, B, C, C_R_, C_L_	<0.001; <0.002 for B	A, A_R_, A_L_	<0.001
B_L_	A, A_R_, A_L_, B, C, C_R_, C_L_	<0.001	A, A_R_, A_L_, C_R_	<0.001; =0.03 for C_R_
C	A, A_R_, A_L_, B_R_, B_L_	<0.001	A, A_R_, A_L_	<0.001
C_R_	A, A_R_, A_L_, B_R_, B_L_	<0.001	A, A_R_, A_L_, B_L_	<0.001; =0.03 for B_L_
C_L_	A, A_R_, A_L_, B_R_, B_L_	<0.001	A, A_R_, A_L_	<0.001

**Table 2:** A comparison of the ROI respiratory magnitudes and ROI-to-TS correlations for men and women: median value (interquartile range). The differences were tested using the Mann–Whitney U-test, with the exact *p*-values listed. Differences found significant at a 5% significance level are printed in bold.

	Median Magnitude/mm, (IQR)		*p*	Median Pearson’s R, (IQR)		*p*
Object	Men	Women		Men	Women	
A	0.92 (0.72)	1.14 (0.74)	0.24	0.71 (0.45)	0.66 (0.34)	0.88
A_R_	0.89 (0.55)	1.22 (0.96)	0.11	0.82 (0.18)	0.74 (0.27)	0.41
A_L_	0.92 (0.73)	1.04 (0.80)	0.08	0.73 (0.36)	0.71 (0.27)	0.54
**B**	**5.20 (2.70)**	**3.81 (1.80)**	**0.02**	0.93 (0.04)	0.94 (0.01)	1
B_R_	2.85 (1.91)	2.41 (0.99)	0.36	0.92 (0.07)	0.93 (0.11)	0.35
**B_L_**	**2.77 (1.51)**	**2.06 (1.12)**	**0.03**	0.90 (0.11)	0.91 (0.09)	0.74
**C**	**6.39 (2.58)**	**5.67 (2.01)**	**0.02**	0.93 (0.06)	0.92 (0.07)	0.81
**C_R_**	**5.59 (2.25)**	**4.40 (2.24)**	**0.02**	0.95 (0.05)	0.93 (0.06)	0.35
**C_L_**	**6.27 (2.08)**	**4.00 (1.96)**	**<0.001**	0.95 (0.05)	0.94 (0.05)	0.27

**Table 3:** **(a)** Correlation between adipose tissue volumes, BMI, patient height and respiratory magnitudes, for different ROIs, for a total of 57 patients. **(b)** Correlation between adipose tissue volumes, BMI, patient height and ROI-to-TS Pearson’s R, for different ROIs, for a total of 57 patients. The heat map indicates negative (blue) and positive (red) correlations, and the intensity of the color is an indicator of the intensity of the correlation, ranging from −1 to +1. Correlations with significance levels of *p* less than 0.05 are listed in bold and considered significant.

**(a)**	ROI magnitude (57 patients)		A	A_R_	A_L_	B	B_R_	B_L_	C	C_R_	C_L_
	SAT%	Spearman’s Rho	−0.17	−0.21	−0.26	**−0.29**	−0.23	**−0.32**	−0.04	−0.23	**−0.28**
		*p*	0.22	0.12	0.05	**0.03**	0.08	**0.02**	0.75	0.08	**0.03**
	VAT%	Spearman’s Rho	**−0.28**	**−0.41**	**−0.41**	0.03	−0.24	−0.04	0.17	−0.03	0.05
		*p*	**0.04**	**0.002**	**0.002**	0.82	0.08	0.77	0.21	0.84	0.71
	TAT%	Spearman’s Rho	−0.26	**−0.39**	**−0.43**	−0.24	**−0.33**	**−0.30**	0.04	−0.21	−0.24
		*p*	0.06	**0.003**	**0.001**	0.07	**0.01**	**0.03**	0.79	0.11	0.08
	BMI	Spearman’s Rho	**−0.31**	**−0.35**	**−0.42**	**−0.28**	**−0.28**	**−0.34**	0.07	−0.21	**−0.27**
		*p*	**0.02**	**0.008**	**0.001**	**0.03**	**0.03**	**0.01**	0.58	0.12	**0.04**
	Patient height	Spearman’s Rho	0.20	0.18	0.15	0.26	0.09	0.09	0.16	0.25	0.17
		*p*	0.12	0.17	0.25	0.05	0.50	0.50	0.23	0.07	0.20
**(b)**	ROI-to-TS correlation (57 patients)		A	A_R_	A_L_	B	B_R_	B_L_	C	C_R_	C_L_
	SAT%	Spearman’s Rho	−0.22	−0.24	−0.26	−0.14	0.05	−0.08	0.01	−0.15	−0.05
		*p*	0.10	0.07	0.05	0.30	0.70	0.54	0.93	0.25	0.73
	VAT%	Spearman’s Rho	**−0.38**	**−0.34**	**−0.34**	−0.11	**−0.33**	**−0.30**	0.23	0.25	0.16
		*p*	**0.004**	**0.01**	**0.01**	0.42	**0.01**	**0.02**	0.09	0.07	0.23
	TAT%	Spearman’s Rho	**−0.35**	**−0.39**	**−0.43**	−0.15	−0.10	−0.16	0.14	0.01	0.05
		*p*	**0.007**	**0.003**	**0.001**	0.27	0.48	0.23	0.32	0.96	0.69
	BMI	Spearman’s Rho	**−0.33**	**−0.31**	**−0.33**	−0.17	−0.13	−0.21	0.17	0.01	0.09
		*p*	**0.01**	**0.02**	**0.01**	0.21	0.32	0.11	0.20	0.90	0.51
	Patient height	Spearman’s Rho	−0.01	−0.11	−0.04	0.22	0.03	0.11	0.09	0.25	0.25
		*p*	0.93	0.39	0.74	0.1	0.85	0.44	0.5	0.07	0.07

Spearman’s Rho = −1 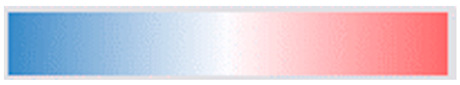 Spearman’s Rho = +1.

## Data Availability

The data presented in this study are stored in a repository at Radiochirurgia Zagreb and are available on request from the corresponding author. The computer code developed for the purposes of conducting this study is available at https://drive.google.com/drive/folders/1HHPEFcJh4UBhZhzL5qzDf7Pc3hItyKyZ (accessed on 8 June 2024).
